# Estimating population size via line graph reconstruction

**DOI:** 10.1186/1748-7188-8-17

**Published:** 2013-07-05

**Authors:** Bjarni V Halldórsson, Dima Blokh, Roded Sharan

**Affiliations:** 1 School of Science and Engineering, Reykjavík University, Menntavegur 1, Reykjavik 101, Iceland; 2 Blavatnik School of Computer Science, Tel Aviv University, Tel Aviv 69978, Israel

**Keywords:** Population size, Haplotypes, Line graphs, Integer programming

## Abstract

**Background:**

We propose a novel graph theoretic method to estimate haplotype population size from genotype data. The method considers only the potential sharing of haplotypes between individuals and is based on transforming the graph of potential haplotype sharing into a line graph using a minimum number of edge and vertex deletions.

**Results:**

We show that the resulting line graph deletion problems are NP complete and provide exact integer programming solutions for them. We test our approach using extensive simulations of multiple population evolution and genotypes sampling scenarios. Our results also indicate that the method may be useful in comparing populations and it may be used as a first step in a method for haplotype phasing.

**Conclusions:**

Our computational experiments show that when most of the sharings are true sharings the problem can be solved very fast and the estimated size is very close to the true size; when many of the potential sharings do not stem from true haplotype sharing, our method gives reasonable lower bounds on the underlying number of haplotypes. In comparison, a naive approach of phasing the input genotypes provides trivial upper bounds of twice the number of genotypes.

## Background

A fundamental problem in population studies is the estimation of the size of the underlying haplotype pool. In these studies, such as genomewide association studies, a number of individuals are genotyped at some single nucleotide polymorphism (SNP) locations. Since we cannot observe the haplotypes of an individual, a common approach to the size estimation problem is to phase the genotype data, i.e., computationally predict the underlying haplotypes. However, haplotype phasing is a notoriously difficult problem [1] and can be optimally solved for small instances only [2].

Here we propose a novel approach that does not require the phasing of the haplotypes. It is based on starting from the easy-to-compute information on potential haplotype sharing between individuals. Specifically, we can test if two individuals have the potential to share a haplotype by checking if their genotypes are consistent with such sharing (i.e., whenever one of the genotypes is homozygous at a certain site, the other is homozygous to the same allele or heterozygous). This information can be encoded in a graph, known as the Clark consistency (CC) graph [3], where each individual (genotype) is represented by a node and an edge is added between two individuals if they can share a haplotype.

The line graph of a graph, is a new graph that has nodes for each edge in the root (original) graph and an edge between two nodes of the line graph if the corresponding edges are adjacent in the root graph. If data were perfect, i.e., the only observed potential sharings were true sharings, then the resulting CC graph would be a line graph whose root graph has haplotypes as nodes and edges connect pairs of haplotypes corresponding to the observed genotypes. The reconstruction of this root graph would then enable us to compute the haplotype population size. In practice, the graph is only close to being a line graph and contains “extra” edges that do not represent true sharing. These extra edges are due to the fact that the genotypes are consistent with each other while there is no common shared haplotypes. For reasonable population structure (as reflected in the simulations presented below), we expect such sharing of genotypes to be rare. Thus, our goal is to find a smallest number of edge deletions that will make the CC graph a line graph. Once we arrive at a line graph we can reconstruct its root graph, thereby getting an estimate of the number of underlying haplotypes, as well as predictions of which individuals share a haplotype (those pairs that remain connected by an edge). We also consider a variant of the line graph deletion problem where node deletions are allowed; those correspond to cases where there are significant genotyping errors or missing data in one of the individuals or genotype pools.

CC graphs may also be determined from data generated using other technologies or experimental protocols, including pooled sequencing [4]. In pooled sequencing, pools of DNA are constructed, each containing the DNA of multiple individuals with each individual typically belonging to multiple pools. Each pool is then genotyped and a conflated genotype for all the individuals in the pool is constructed. To create a CC graph we add an edge between two individuals if they can share a haplotype, that is, if for every pair of pools containing these individuals, one in each pool, the two pools are consistent with sharing a haplotype.

We study the complexity of the resulting line graph modification problems. We observe that both problems (edge- and vertex-deletion) are NP-complete and provide polynomial integer programming formulations to solve them. We also show how the method can be used as a first step in an iterative algorithm for haplotype phasing. We test our approach using extensive simulations of multiple population evolution and genotypes sampling scenarios and on data from the International HapMap Consortium [5]. Our computational experiments show that when most of the sharings are true sharings the problem can be solved very fast and the estimated size is very close to the true size. In cases where many of the potential sharings do not stem from true haplotype sharing, due to spurious edge or node insertions, our method gives reasonable lower bounds on the underlying number of haplotypes. In comparison, a naive approach of phasing the input genotypes provides trivial upper bounds of twice the number of genotypes.

In the remainder of the paper, we start by presenting the background for this paper, the definition of our optimization problems and an analysis of their complexity. We then present five different integer programming formulations to solve our optimization problems. Finally, we perform computational experiments on both simulated data and on data from the International HapMap Consortium.

## Preliminaries

Let *G* = (*V*,*E*) be a graph with a set *V* of *n* vertices and a set *E* of *m* edges. The line graph of *G*, denoted by *L*(*G*), is constructed by having a node in *L*(*G*) for each edge in *G* and an edge between two nodes of *L*(*G*) if the corresponding edges are adjacent in *G*.

### Line graph and CC graphs

If *L*(*G*) is the line graph of *G*, we refer to *G* as the root graph of *L*(*G*). Line graphs have been studied by a number of authors. Whitney [6] showed that, apart from a single exception, the root graph of a line graph is unique. Lehot [7] and Roussopoulos [8] have independently given linear-time algorithms for detecting whether a graph is a line graph and outputting its root graph. Van Rooij and Wilf [9] gave a characterization of line graphs in terms of nine forbidden subgraphs. The characterization can be stated as follows; A triangle in a graph is even if every other node is adjacent to 0 or 2 nodes in the triangle; it is odd otherwise. A graph is a line graph iff it contains no two odd triangles that share an edge is claw-free (a claw is a node connected to three nodes not connected to each other). This characterization can also be stated as a list of nine distinct subgraphs that are forbidden from the line graph. Each one of the nine different subgraphs has at most six nodes and ten edges.

A key component of our approach is the graph of potential sharing of haplotypes between individuals. This graph, called the Clark consistency (CC) graph, was first suggested in the context of a method for haplotype phasing by Andrew Clark [10]. Given the genotypes of a set of individuals, the CC graph has one node for each individual and an edge between two individuals if their genotypes are consistent with sharing a haplotype, i.e., if for every site where one of the individuals is homozygous, the other individual is either homozygous for the same allele or heterozygous. As the haplotypes of individuals that are homozygous for the whole region being considered are easily determined, we assume that every given individual has two different haplotypes (i.e., it is heterozygous for at least one of the genotyped markers) and no two individuals have the same pair of haplotypes.

As we show below, CC graphs and line graphs are closely related. Let *H* be the CC graph of a given set of genotypes. We say that *H* is allelable if there is an assignment of pairs of colors to its nodes so that every two adjacent nodes share exactly one color and every two non-adjacent nodes have distinct color sets. Under this assignment every color represents a haplotype or an allele in the population. The number of colors is the estimated number of genotypes. The following observation stands at the basis of our computational approach.

#### 

**Lemma 2.1.** *H* is allelable iff it is a line graph.

#### 

*Proof.* If *H* is allelable then construct a graph *G* in which every allele of *H* represents a node and edges connect alleles that are paired together in the nodes of *H*. It is easy to see that *H* is the line graph of *G*.

Conversely, if *H* = *L*(*G*) is a line graph then one can assign distinct labels to the nodes of the corresponding root graph *G* and use these labels to label the edges of *G* and, hence, the nodes of *H*. This assignment clearly shows that *H* is allelable. □

Lemma 2.1 implies that a set of haplotypes providing a valid phasing of genotypes will always form a line graph. The converse, however, need not be true as the SNP data of the original genotypes are not encoded in our formulation.

### Problem definition and its complexity

As the input data may have more edges than the underlying line graph, we do not expect real data to yield line graphs. Rather it is desirable to transform a given CC graph into a line graph using a minimum number of node and edge deletions. In the following we formulate three versions of the problem.

#### 

**Problem 2.1.** Given a graph *G* = (*V*,*E*), find a line graph $G^{\prime }=(V,E^{\prime })$ such that $E^{\prime }\subseteq E$ and $|E-E^{\prime }|$ is minimized.

#### 

**Problem 2.2.** Given a graph *G* = (*V*,*E*), find a line graph $G^{\prime }=(V^{\prime },E)$ such that $V^{\prime }\subseteq V$ and $|V-V^{\prime }|$ is minimized.

We also consider a combined problem where both nodes and edges may be removed from a graph:

#### 

**Problem 2.3.** Given a graph *G* = (*V*,*E*) and a constant *α*, find a line graph $G^{\prime }=(V^{\prime },E^{\prime })$ such that $E^{\prime }\subseteq E$, $V^{\prime }\subseteq V$ and $|E-E^{\prime }|+\alpha |V-V^{\prime }|$ is minimized.

Yannakakis [11] has shown that the problems of deleting a minimum number of edges or nodes of a graph in order to make it into a line graph are both NP-hard.

As the problems can be formulated as a problem of avoiding particular subgraphs which have at most 6 nodes and 10 edges, by [12] both problems are fixed parameter tractable and can be solved in time max{*m*,*n*}*O*(10^*i*^) and max{*m*,*n*}*O*(6^*j*^), respectively, where *i* (resp., *j*) is the minimum number of edges (resp., vertices) that need to be deleted. Using the techniques of Niedermeier and Rossmanith [13], the node deletion variant can be solved in max{*m*,*n*}*O*(5.19^*j*^) time.

Problem 2.2 can be formulated as a hitting set problem, where all the sets that need to be hit are of size 4,5 or 6. Trevisan [14] showed that the hitting set problem, when the size of the input sets is bounded by *B* ≥ 2 (*B* = 6 in our case), is hard to approximate to within $B^{-\frac{1}{19}}$. The fact that all sets are of size at most six also implies a 6-approximation algorithm for the line graph node deletion problem [15].

## Integer programming formulation

Here we provide integer programming formulations for Problems 2.1, 2.2 and 2.3. The formulations rely on the characterization given in Lemma 2.1. We let [*i*,*j*] represent {*i*,*i* + 1,...,*j*}.

### Edge deletions

Let *G* = (*V*,*E*) be the input CC graph. Our basic program has the following sets of binary variables: (i) A variable *d*_*e*_ for every edge *e* ∈ *E*, where *d*_*e*_ = 1 iff *e* is deleted. (ii) A variable *x*_*v*,*j*_ for every node *v* ∈ *V* and every possible allele *j* ∈ [1,2*n*], where *x*_*v*,*j*_ = 1 iff individual *v* has allele *j*. (iii) A variable *s*_*e*,*j*_ for every edge *e* ∈ *E* and every possible allele *j* ∈ [1,2*n*], where *s*_*e*,*j*_ = 1 iff the two endpoints of *e* share allele *j*. It is formulated as follows:

$$\begin{array}{llll} \text{min} & \underset{e}{\sum }d_{e} & & \\ \mathrm{s.t.} & \overset{2n}{\underset{j=1}{\sum }}x_{v,j}=2 & \forall v\in V & \\ & x_{v,j}+x_{u,j}\leq 1 & \forall (u,v)\notin E,j\in \;[1,2n] & \\ & \overset{2n}{\underset{j=1}{\sum }}s_{e,j}=1-d_{e} & \forall e\in E & \\ & x_{u,j}+x_{v,j}-1\leq s_{e,j} & \forall e=(v,u)\in E,j\in \;[1,2n] & \\ & s_{e,j}\leq x_{u,j} & \forall j\in \;[1,2n],u\in e & \\ & d_{e},x_{v,j},s_{e,j}\in \{0,1\} & \forall e\in E,v\in V,j\in \;[1,2n] & \end{array}$$

In this formulation, the first constraint ensures that each node is assigned two distinct colors; the next two constraints ensure that non-adjacent nodes (before or after edge deletion) do not share a color; and the fourth and fifth constraints ensure that the edge sharing variables are consistent with the node coloring variables. A potential problem with the formulation, however, is that different permutations of the colors yield equivalent solutions. To tackle this problem, we use the symmetry breaking techniques of [16]. Specifically, we start by ordering the nodes from 1 through *n*. Each node “owns” two colors: node *i* owns colors (2*i* - 1) and 2*i*. The fact that *x*_*v*,*j*_ = 1, where *j* = 2*v* - 1 or *j* = 2*v* means that *v* is the first node to use color *j*. *v* is required to use color 2*v* - 1 before it uses color 2*v*.

Overall, node *v* has access to colors 1,2,…,2*v* and can use some of the previously used colors or be the first ordered node to use new colors. These restrictions can be represented by the following additional constraints:

$$\begin{array}{llll} x_{v,2v}\leq x_{v,2v-1} & \forall v\in \;[1,n] & & \\ x_{j,2i-1}\leq x_{i,2i-1} & \forall j\in \;[i+1,n] & & \\ x_{j,2i}\leq x_{i,2i} & \forall j\in \;[i+1,n] & & \end{array}$$

We observe that a number of the variables in the above formulation can be automatically set to 0 and removed from the formulation. In particular, if (*i*,*j*) ∉ *E*,*i* < *j* then *x*_*j*,2*i*_ = *x*_*j*,2*i* - 1_ = 0, as *j* and *i* cannot share a color when they do not share an edge. If *e* = (*u*,*v*), we also note that *s*_*e*,*j*_ = 0 unless both *u*,*v* can be colored with color *j*, that is if $(v,\left\lfloor \frac{j+1}{2}\right\rfloor ),(u,\left\lfloor \frac{j+1}{2}\right\rfloor )\in E$.

### Node deletions

If a graph does not contain one of the forbidden substructures then the graph is allelable. Otherwise, a vertex has to be removed from each of the forbidden subgraphs. Our algorithm relies on listing all occurrences of the forbidden substructures and then solving the hitting set problem on the set of forbidden substructures.

We observe that the deletion of a node will not create a new forbidden substructure. The node deletion problem can thus be formulated as an integer program by listing all forbidden substructures and removing one node from each one of them. We let *t*_*n*_ be a binary variable representing whether node *i* is removed and denote by F the set of all forbidden induced subgraphs of a line graph. Then the program is formulated as follows:

$$\begin{array}{llll} \text{min} & \underset{n}{\sum }t_{n} & & \\ & \underset{n\in F}{\sum }t_{n}\geq 1 & \forall F\in F & \\ & t_{n}\in \{0,1\} & \forall n\in V & \end{array}$$

### Edge and node deletions

In real data, both genotype errors and spurious sharing relations may happen at the same time, leading to a combined node- and edge-deletion problem. We define *d*,*x*, *s* and *t* as before. We define the variable *r*_*e*_ as a boolean variable representing that an edge has been removed, which occurs when either one of its adjacent nodes or the edge itself is deleted. We let *α* be the relative cost of node deletion with respect to edge deletion. The combined program is as follows:

$$\begin{array}{llll} \text{min} & \underset{e}{\sum }d_{e}+\alpha \underset{n}{\sum }t_{n} & & \\ \mathrm{s.t.} & \overset{2n}{\underset{j=1}{\sum }}x_{v,j}=2-2t_{v} & \forall v\in V & \\ & x_{v,j}+x_{u,j}\leq 1 & \forall (u,v)\notin E,j\in \;[1,2n] & \\ & \overset{2n}{\underset{j=1}{\sum }}s_{e,j}=1-r_{e} & \forall e\in E & \\ & x_{u,j}+x_{v,j}-1\leq s_{e,j} & \forall e=(v,u)\in E,j\in \;[1,2n] & \\ & s_{e,j}\leq x_{u,j} & \forall j\in \;[1,2n],u\in e & \\ & r_{e}\leq t_{v}+t_{u}+d_{e} & \forall e=(u,v)\in E & \\ & d_{e}\leq r_{e} & \forall e\in E & \\ & t_{u}\leq r_{e} & \forall u\in e,e\in E & \\ & d_{e},t_{v},x_{v,j},s_{e,j},r_{e}\in \{0,1\} & \forall e\in E,v\in V,j\in \;[1,2n] & \end{array}$$

We further augment this integer program with the symmetry breaking techniques described in Section ‘Edge deletions’.

### Edge deletions with errors

The model described in the previous sections does not deal with noise due to genotype miscalls. Genotyping errors may lead to haplotypes not sharing an edge when they in fact should. There are two ways in which this problem can be handled by our framework. The first method that we suggest is to add edges when the genotypes are compatible in all but a fixed number of error locations, our formulation would then be to remove the minimum number of edges, possibly with weights related to the number of errors in the edges.

The second allows for edge insertions, possibly with weights related to the number genotyping errors that are needed to have occurred for there to be an edge between the two nodes. We describe below an extension of our integer programming formulation to allow for such edge insertions, solving the more general problem of editing a graph (by edge insertions and deletions) to form a line graph.

As before, we let *G* = (*V*,*E*) be a graph which we would like to make allelable with *n* = ∣ *V* ∣ and *m* = ∣*E*∣. We introduce *b*_*e*_ as an indicator for an edge *b*_*e*_ = 1 if *e* ∈ *G* and *b*_*e*_ = 0 if *e* ∉ *G*. We introduce a variable *c*_*e*_ for every edge *e* ∉ *G*, we let *c*_*e*_=1 if *e* is inserted and *c*_*e*_ = 0 if *e* is not inserted. We let *w*_*e*_ denote a weight associated with each edge that can be deleted or inserted. We let the variables *x*_*v*,*j*_vand *d*_*e*_ have the same interpretation as before. The variables *s*_*e*,*j*_ also have the same interpretation as before, except that *e* can now be any pair *e* = (*u*,*v*) ∈ *V* × *V*,*u* ≠ *v*.

$$\begin{array}{llll} \text{min} & \underset{e\in E}{\sum }w_{e}d_{e}+\underset{e\in V\times V-E}{\sum }w_{e}c_{e} & & \\ \mathrm{s.t.} & \overset{2n}{\underset{j=1}{\sum }}x_{v,j}=2 & \forall v\in V & \\ & x_{v,j}+x_{u,j}\leq 1+b_{e}-c_{e} & \forall (u,v)\in V\times V,v\neq u,j\in \;[1,2n] & \\ & \overset{2n}{\underset{j=1}{\sum }}s_{e,j}=b_{e}+c_{e}-d_{e} & \forall e\in V\times V & \\ & x_{u,j}+x_{v,j}-1\leq s_{e,j} & \forall e=(v,u)\in V\times V,v\neq u,j\in \;[1,2n] & \\ & s_{e,j}\leq x_{u,j} & \forall j\in [1,2n],u\in e & \\ & c_{e},d_{e},x_{v,j},s_{e,j}\in \;\{0,1\} & \forall v\in V,e\in E,j\in \;[1,2n] & \end{array}$$

This formulation can be extended to capture both the symmetry breaking constraints and the node deletion case, described earlier.

### Haplotype phasing under the parsimony criteria

Researchers are frequently interested in knowing not only the number of haplotypes for a particular instance but also the haplotypes themselves. While the main purpose of the paper is not to present a method for haplotype phasing, we will show how the method presented here can be used as a first step in a constraint generation approach for haplotype phasing.

In order to determine the minimum number of haplotypes we would start by solving a modified version of our integer program for edge deletions. We exploit the symmetry breaking constraints to count and minimize the number of haplotypes used to explain the line graph.

$$\begin{array}{llll} \text{min} & \underset{v\in V}{\sum }x_{v,2v-1}+x_{v,2v} & & \\ \mathrm{s.t.} & \overset{2v}{\underset{j=1}{\sum }}x_{v,j}=2 & \forall v\in V & \\ & x_{v,j}+x_{u,j}\leq 1 & \forall (u,v)\notin E,j\in \;[1,2n] & \\ & \overset{2n}{\underset{j=1}{\sum }}s_{e,j}\leq 1 & \forall e\in E & \\ & x_{u,j}+x_{v,j}-1\leq s_{e,j} & \forall e=(v,u)\in E,j\in \;[1,2n] & \\ & s_{e,j}\leq x_{u,j} & \forall j\in \;[1,2n],u\in e & \\ & x_{v,2v}\leq x_{v,2v-1} & \forall v\in \;[1,n] & \\ & x_{j,2i-1}\leq x_{i,2i-1} & \forall i\in \;[1,n],j\in \;[i+1,n] & \\ & x_{j,2i}\leq x_{i,2i} & \forall i\in \;[1,n],j\in \;[i+1,n] & \\ & x_{v,j}\in \{0,1\} & \forall v\in V,j\in \;[1,2v] & \\ & s_{e,j}\in \{0,1\} & \forall e\in E,j\in \;[1,2n] & \end{array}$$

We note that as there is no cost associated with deleting edges we no longer have a need for the *d*_*e*_ variables.

We note that the line graph minimizing the number of haplotypes will always provide a lower bound on the number of haplotypes. There however may be other constraints provided by the genotypes that will not be fulfilled by the above optimization formalization. The next step in our algorithm is to search for sets of haplotype sharings inconsistent with the solution found by the integer program.

We let *I* be the solution of the integer program and let *e*∈*I* denote that in *I* there exists a color *j* such that *s*_*e*,*j*_=1 We start by initializing a set *S* with the two nodes of an edge *e*∈*I*. We then perform a partial phasing of the genotypes of these individuals. In the partial phasing we consider each SNP where one of the individuals is homozygote and the other is heterozygote; the shared haplotype must then contain the allele of the heterozygote.

We then grow *S* by selecting a neighbor, *v* to one of the nodes in *S* and add *v* to *S*. If no such neighbor exists then we have a valid partial phasing for the nodes of *S* and the nodes in *S* need not be considered further. If such a neighbor does exist, then we use *S* to partially phase the genotype of *v*. As we describe below, such a phasing of *v* may not always be possible, if it is we update the haplotypes in *S* and iterate the growth of *S*. If such a phasing does not exist we conclude that not all of the neigbor relations considered so far can hold simultaneously and we resolve the integer program with the added constraint:

$$\underset{e\in I}{\sum }\overset{n}{\underset{j=1}{\sum }}s_{e,j}\leq |\{e|e\in I\cap S\}|-1$$

We now describe how we use *S* to partially phase *v*. We let *h*_*u*_ (*v*) be the haplotype *u* shares with *v*. This haplotype is uniquely defined; At the time *v* is added to *S*, *u* has two partially phased haplotypes, at least one of which is shared with a neighbor *w* ∈ *S*. If *I* indicates that all three of *u*,*v* and *w* share a haplotype then the haplotype *v* shares with *u* is the haplotype *u* shares with *w*, if not *u* shares with *v* the haplotype it does not share with *w*. It is possible that *h*_*u*_ (*v*) has been phased for one allele of a SNP while *v* is homozygote for the other allele, in this case *v* cannot be phased consistently with the partial phasing of *S*. If not, we add constraints from *v* to the phasing of haplotypes of *S*; If *h*_*u*_ (*v*) is heterozygote for a SNP and *v* is homozygote then *h*_*u*_ (*v*) must at that SNP be assigned the allele of *v* and the other haplotype of *u* must be assigned the other allele. As the nodes of *S* are connected the fact that *v* is heterozygote for the SNP will affect the partial phasings of all nodes in *S*. Finally, we note that *v* may be connected to more than one node in *S*, we similarly consider whether *v* is consistent with the partial phasing of those nodes.

## Experimental results

We comprehensively test our algorithm for deleting edges and vertices on data from the International HapMap Consortium [5] and under two simulated scenarios, corresponding to a bottleneck population isolate and a population that has continuously undergone recombination and mutation. For the population isolate we show that we can almost fully recover the haplotype structure from the sharing information alone. For the population that has undergone continuous recombination and mutation we get a bound on the number of haplotypes that is close to the true number of haplotypes in the population. Our experiments further reveal that the occurrence of genotypes showing a large degree of sharing does not materially affect our ability to estimate the number of haplotypes in the remaining population. The computational experiments were done using CPLEX 12, making use of a single CPU processor core with 4GB of memory.

### Bottleneck population

Haplotypes that are shared across a long region are with high probability identical by descent, i.e. they are the result of the genetic material of a single forefather being passed to a number of his descendents. Some of the haplotypes, however, will be identical by state only, i.e., the haplotypes are the same but cannot be traced to a single common forefather. The probability of identical by state sharing decreases rapidly with the length of the haplotype being considered. The probability of identity by state sharing depends on the size of the ancestral population. We simulate graphs that might occur from the detection of identity by state sharing.

We use Hudson’s ms simulator [17] to simulate realistic genotype populations. We assume that there is an initial small bottleneck population that then rapidly expanded. We simulate genotypes for the initial population and then generate the current population as a random sample with replacement from this initial population. The parameters for the simulation are chosen such as to sample approximately a 5 cM locus, or 3027 SNPs, a size for which it is reasonable to expect that no recombinations would take place in the region between two individuals being studied. We consider two sets of experiments for this scenario. In the first experiment we vary the size of the population but keep the number of times that each haplotype is sampled on expectation fixed. In the second experiment we vary the expected number of times that each haplotype is sampled and keep the size of the population fixed.

In Table 1 we fix the expected number of times that a haplotype is sampled as 1 and we vary the size of the population, which we present as the number of haplotypes in the ancestral population. It can be seen that the number of edges grows roughly linearly with the size of the population being sample. Our simulations show that the number of estimated haplotypes is in close agreement with the true number of haplotypes in the sample. As some of the haplotypes are sampled multiple times the number of haplotypes in the sample is typically smaller than the actual population size. Notably, all instances are solved very fast (less than a second).

**Table 1:** Effect of increasing population size

**Genotypes**	**# edges**	**# edges**	**Estimated #**	**True #**	**Compute**
**sampled**		**removed**	**haplotypes**	**haplotypes**	**time (s)**
25	21	0	33	33	0
50	46	1	64	63	0.04
75	74	0	95	95	0.01
100	105	0	125	126	0.02
125	129	2	154	153	0.05
250	294	1	300	300	0.07
500	634	3	589	594	0.29

Genotypes are generated by sampling with replacement two haplotypes at a time from a population of size twice the number of genotypes. The size of this population is varied. The estimated and true numbers of haplotypes both refer to the set of haplotypes underlying the sampled genotypes.

In Table 2 we look at an initial bottleneck population of 100 haplotypes while varying the number of genotypes sampled from this population from 25 to 125. This implies that each haplotype in the ancestral population is sampled between 0.5 and 2.5 times (on average). As the sample size increases, our estimate of the number of haplotypes tightly follows the true number of haplotypes sampled from the population, with both approaching the actual population size of 100. We observe that the number of edges in the graph grows roughly as the square of the sample size. The CPU time used for all these instances is minimal.

**Table 2:** Effect of increasing coverage

**# Genotypes**	**Coverage**	**# edges**	**# edges**	**Estimated #**	**True #**	**Compute**
			**removed**	**haplotypes**	**haplotypes**	**time (s)**
25	0.5	17	0	37	37	0.00
50	1	46	1	64	63	0.04
75	1.5	98	0	82	82	0.04
100	2	186	0	87	88	0.02
125	2.5	301	0	92	92	0.09

Performance evaluation with respect to a sample drawn from an initial population of 100 haplotypes. The number of individuals drawn from the sample is varied.

### Recombinant population

The second scenario that we simulate is a population that is undergoing mutation and recombination. We fix mutation rate at 10^-9^ per generation and recombination rate between two adjacent base pairs at 10^-8^ per generation. In all of our experiments we simulate a 1MB region, varying the number of individuals in the ancestral population (*N*_0_) and the number of individuals sampled in the current population.

First, we vary the size of the initial population, while leaving the number of individuals considered mostly constant (see Table 3). We observe that when the size of the initial population grows the probability that two individuals can share a haplotype decreases rapidly. Many of the edges observed in these graphs are due to sharing between genotypes that are not due to the sharing of haplotypes. The graphs being considered are therefore far from being line graphs and many edges need to be removed in order to make them into ones. Nevertheless, we are able to give estimates of the number of haplotypes in a population even in this setting.

**Table 3:** Effect of population size in a recombinant population

***N***_***0***_	**# SNPs**	**# genotypes**	**# edges**	**# edges**	**Estimated #**	**True #**	**Compute**
				**removed**	**haplotypes**	**haplotypes**	**time (s)**
500	116	100	484	245	83	144	1216
1000	231	100	235	105	114	172	120
2500	584	100	23	0	178	189	0.0
5000	1215	100	15	0	185	195	0.0
10000	3027	500	135	1	880	894	5

Performance evaluation for a recombinant population while varying population size.

The quality of our estimate is, not surprisingly, dependent on the number of edges that need to be removed to create a line graph. Our estimated number of haplotypes is consistently smaller than the true number of haplotypes, but the number of estimated haplotypes is never below 57% of the true number of haplotypes. Similar conclusions can be drawn from the second set of experiments in which we fix the size of the initial population and vary the size of the sample that we draw from that population (see Table 4).

**Table 4:** Effect of increasing coverage in a recombinant population

***N***_***0***_	**# SNPs**	**# genotypes**	**# edges**	**# edges**	**Estimated #**	**True #**	**Compute**
				**removed**	**haplotypes**	**haplotypes**	**time (s)**
1000	193	50	38	5	73	87	5
1000	231	100	235	105	114	172	120
1000	289	200	738	374	170	287	300

Performance evaluation on a recombinant population while varying sample size.

### Combined node and edge deletions

We simulate a scenario in which there are genotypes showing a high degree of excess sharing of haplotypes. To this end, we focus on the data set of 50 genotypes presented in Table 4, and designate increasing subsets of the genotypes as being partially observed, i.e., only a subset of their markers is observed supposedly due to failure or otherwise missing data.

In more detail, in Table 5 we modify a graph consisting of 50 genotypes, consisting of 38 edges where 5 edges have to be removed to transform the graph into a line graph. We let 5,10 or 15 of the genotypes be partially observed, i.e., genotyped at only at 30% of their markers. We show the number of edges added to the graph due to these partial observations. Next, we apply our combined node and edge deletion algorithm and use two settings for the alpha parameter controlling the relative penalty of node vs. edge deletion. The first value that we use, *α* = 1.5, prefers a node deletion whenever more than one of the node’s adjacent edges has to be deleted. The second value of 4 allows up to 4 edge deletions before the node deletion is preferred. In each setting we provide the the number of edges and genotypes removed and the number of removed genotypes that were partially observed. We evaluate the size estimate given by the algorithm against the true number of haplotypes in the data set without counting the haplotypes in the partially observed genotypes.

**Table 5:** Partial genotypes

**# Partial**	**# Extra**	***α***	**# genotypes**	**# partial genotypes**	**# edges**	**Estimated #**	**True #**	
**genotypes**	**edges**		**removed**	**removed**	**removed**	**haplotypes**	**haplotypes**	
5	40	1.5	5	4	1	68	78	
5	40	4	2	2	8	68	78	
10	70	1.5	10	9	1	63	71	
10	70	4	4	3	18	63	71	
15	102	1.5	13	9	0	55	62	
15	102	4	7	6	14	59	62	

Performance evaluation in a population where some of the genotypes are only partially observed.

We observe that the occurrence of the partial genotypes does not appear to materially affect our estimate of the number of haplotypes in the remaining population. We also observe that the largest number of nodes removed in each experiment are from the subset corresponding to the partially observed genotypes. When *α* = 1.5, 60 - - 90*%* of the partially observed genotypes are removed and less than 12% of the other genotypes. When *α* = 4, 30 - - 40*%* of the partial genotypes are removed and less than 3% of the other genotypes. All computations presented in Table 5 finished in under one second.

### Comparison to a phasing-based estimation

Apart from the preprocessing, the complexity of our method does not depend on the number of genotyped markers. In contrast, many haplotype phasing methods are not able to handle the large number of markers dealt with in our approach [2]. We thus opted to compare our method to phasing-based estimates derived from the application of the BEAGLE phaser [18] to our data. Surprisingly, in all the simulated settings, the number of haplotypes estimated by BEAGLE was twice the number of genotypes, which is a trivial upper bound for the number of haplotypes in a population.

### Number of alleles in HapMap populations

We use data from seven of the HapMap [5] populations (cf. Table 6), not considering those populations that contain trios, as sharing of haplotypes between trios is to be expected, which would skew our results. As the size of the populations is variable and in order to have comparable results between populations, we select a sample of 77 individuals from each of these populations. We select regions of sizes 200 and 400 SNPs and compute the haplotype sharings over those regions. We run ten different data sets for each population, selected as the first ten non-overlapping windows on chromosome 1, for a total of 140 data sets. Most of the computations finished in less than one second, a few however took considerable longer time to solve and six of the simulations were stopped after having run without producing an optimal solution after 24 hours. We present results for the remaining 134 instances in Tables 7 and 8.

**Table 6:** Hapmap populations

**Abbreviation**	**Description**
ASW	African ancestry in Southwest USA
CHB	Han Chinese in Beijing, China
CHD	Chinese in Metropolitan Denver, Colorado
GIH	Gujarati Indians in Houston, Texas
JPT	Japanese in Tokyo, Japan
LWK	Luhya in Webuye, Kenya
TSI	Toscani in Italia

A list of the HapMap populations used in this study and their abreviations.

**Table 7:** Hapmap populations

**Population**	**Number of edges**	**Number of haplotypes**	**Number of edges deleted**
ASW	121.6 (58–313)	94.3 (70–111)	38.2 (4–161)
CHB	247.1 (54–758)	94.8 (72–118)	90.9 (14–209)
CHD	265.2 (27–811)	99.9 (70–132)	74.8 (5–189)
GIH	193.3 (72–373)	91.7 (57–119)	86.1 (23–188)
JPT	277.5 (38–812)	90.0 (69–131)	102 (13–179)
LWK	105.8 (28–244)	104.7 (80–129)	33.1 (2–98)
TSI	267.6 (79–579)	92.4 (69–108)	94.0 (20–225)

The number of haplotypes estimated in a sample of 77 individuals when considering haplotypes of length 200 SNPs. Number given are the average (min- max) of 10 different datasets.

**Table 8:** Hapmap populations

**Population**	**Number of edges**	**Number of haplotypes**	**Number of edges deleted**
ASW	57 (48–73)	110.1 (102–115)	1.7 (0–9)
CHB	35.7 (0–106)	132.3 (103–154)	10.2 (0–42)
CHD	46.9 (1–141)	129.7 (100–153)	15.8 (0–65)
GIH	41.1 (13–95)	126.5 (109–143)	8.6 (0–36)
JPT	60 (4–158)	122.7 (92–151)	21.3 (0–66)
LWK	37.5 (11–97)	129.8 (119–143)	1.6 (0–6)
TSI	41.2 (4–115)	130.7 (105–150)	12.9 (0–46)

The number of haplotypes estimated in a sample of 77 individuals when considering haplotypes of length 400 SNPs. Number given are the average (min-max) of 10 different datasets.

We run our edge deletion algorithm on each one of the populations to estimate the number of haplotypes. This estimate can be expected to correlate with the effective population size for the populations; Populations with a smaller effective populations size can be expected to have a higher number of haplotype sharings and fewere haplotypes in our samples.

We observe that the number of haplotype sharings is smaller in the two populations of African heritage (ASW and LWK) than the non-African populations. This indicates that these populations have higher genetic diversity and is consistent with the “out of Africa” theory. An exception to this rule is that when considering regions of 400 SNPs, the ASW populations has a higher average amount of haplotype sharings than some of the other populations. When looking more closely at the data we observed that this higher rate of haplotype sharing is in large part due the same pairs of individuals sharing haplotypes across all 10 datasets, indicating an identical by descent sharing among those pairs of individuals. We further observe that the ASW population has the fewest number of haplotypes when considering regions of 400 SNPs and that those solutions are found while deleting very few edges.

## Conclusions

We show that the problem of assigning alleles to individuals when only information about the sharing of alleles between individuals is known is equivalent to the problem of determining whether a graph is a line graph. When sharing information is not perfect we give polynomial size integer programming algorithms for determining allele sharing. We show how this method can be used to estimate the genetic diversity of different populations. We suspect that the method proposed here may be useful in computing important parameters in population genetics such as the effective population size, however a careful analysis of its statistical properties is necessary.

We further show how this method can be extended to give an iterative optimization algorithm for haplotype phasing. We demonstrate that the first iteration in this algorithm can solve larger problem instances that can in general be solved using common implementations of the haplotype phasing algorithm. As we have not provided an implementation of a haplotype phasing algorithm it remains to be seen if our proposed algorithm is practical and effective.

## Competing interests

The authors declare that they have no competing interests.

## Authors’ contributions

Methodology was developed by BVH and RS. Experiments were run by DB. Manuscript was written by BVH, DB and RS. All authors read and approved the final manuscript.
